# Understanding stakeholder perspectives on integrating and sustaining a vertical HIV prevention programme into routine health services in Zimbabwe: a qualitative study

**DOI:** 10.1136/bmjgh-2024-018732

**Published:** 2025-08-11

**Authors:** Amanda Marr Chung, Joseph Murungu, Peter Case, Precious Chitapi, Rudo Chikodzore, Jonathan Gosling, Sinokuthemba Xaba, Getrude Ncube, Owen Mugurungi, Patience Kunaka, Ndola Prata, Roly Daniel Gosling, Stefano M Bertozzi, Colette Auerswald

**Affiliations:** 1 Center for Innovation in Global Health, Stanford University, Stanford, California, USA; 2 Institute for Global Health Sciences, University of California San Francisco, San Francisco, California, USA; 3 HEALTHQUAL, University of California San Francisco, Harare, Zimbabwe; 4 Bristol Business School, University of the West of England, Bristol, UK; 5 School of Health Systems and Public Health, University of Pretoria, Pretoria, South Africa; 6 College of Business, Law & Governance, James Cook University, Townsville City, Queensland, Australia; 7 Precious Innovations, Harare, Zimbabwe; 8 United Nations Children’s Fund Zimbabwe, Harare, Harare Province, Zimbabwe; 9 Ministry of Health and Child Care, Gwanda, Matebeleland South, Zimbabwe; 10 Pelumbra Limited, Exeter, UK; 11 Ministry of Health and Child Care, Harare, Zimbabwe; 12 Bixby Center for Population, Health and Sustainability, University of California Berkeley, Berkeley, California, USA; 13 Epidemiology and Biostatistics, University of California San Francisco, San Francisco, California, USA; 14 Department of Disease Control, London School of Hygiene & Tropical Medicine, London, UK; 15 School of Public Health, University of California Berkeley, Berkeley, California, USA; 16 Global Health, University of Washington School of Public Health, Seattle, Washington, USA; 17 Instituto Nacional de Salud Pública, Cuernavaca, México

**Keywords:** Global Health, Health Personnel, Health systems, HIV, Zimbabwe

## Abstract

**Introduction:**

The transition of voluntary medical male circumcision (VMMC), an HIV prevention service, in Zimbabwe from a donor-funded to a government-owned programme involves the collective efforts and alignment of national and subnational government leaders, managers, healthcare providers, village health workers, community members, donors and implementing partners. We sought to understand stakeholders’ perspectives on barriers, facilitators and recommendations as a vertical HIV prevention programme transitioned to an integrated, government-led model.

**Methods:**

We conducted 54 semistructured stakeholder interviews at the national and subnational levels. Interviews were audio recorded, transcribed and thematically analysed.

**Results:**

Participants highlighted a range of psychological and structural barriers and facilitators to integrating and sustaining the VMMC programme. Respondents mentioned financing and staffing barriers to integration, particularly a lack of domestic resources, the transition from a fee-for-service to a facility-based performance model and staff attrition. Notably, resistance to changing the VMMC programme’s operations was a significant barrier that may be tied to individual psychological barriers such as loss of power and job security. Donors and partners continued to control the funding for VMMC. Ideally, the Ministry of Health and Child Care should have more autonomy over these decisions. At the subnational level, there is an opportunity for increased responsibility and a greater sense of ownership through the decentralisation of governance.

**Conclusions:**

To ensure successful integration and local ownership of VMMC as an HIV prevention programme, stakeholders must address both psychological and structural barriers while aligning their perspectives on the transition. Individual providers have valid concerns about their financial security and the burden of additional responsibilities without adequate compensation. It is crucial for donors and partners to reduce their involvement and oversight. Additionally, resolving the financial barriers that prevent the government from having complete control of the programme will require empowering local government stakeholders to fully take ownership.

**WHAT IS ALREADY KNOWN ON THIS TOPIC**

- Programme sustainability and integration represent critical global health challenges, particularly in light of the increasing funding gap in development assistance for health following recent withdrawal of support from foreign governments. Achieving the Sustainable Development Goals and delivering basic health services are both at risk.
- For voluntary medical male circumcision (VMMC), a vertical health programme funded by external donors, countries need to plan for integration and sustainability of the programme to maintain epidemiological benefits, such as reduced HIV transmission, after donor support has been withdrawn.
- Few studies describe national and subnational level stakeholder perspectives on the barriers, facilitators and recommendations that contribute to the successful transfer of ownership and integration of a large-scale, donor-funded vertical health programme into government services.

**WHAT THIS STUDY ADDS**

- Sustainability of the HIV prevention programme is hindered by insufficient domestic financing and fragmented budget management. Transitioning to government ownership and financing is a long-term goal complicated by economic instability, competing health priorities and limited domestic resources.
- High staff attrition, driven by low wages and poor working conditions, disrupts service delivery and erodes institutional knowledge. Addressing these issues with better remuneration, non-financial incentives and training could improve retention and motivation.
- Decentralising VMMC to primary healthcare facilities faces challenges such as limited space, staff shortages and confidentiality concerns. Overcoming these barriers could improve accessibility, trust and timeliness of services, making the programme more patient-centred.
- Power asymmetry between donors and government limits local ownership and integration of the HIV prevention programme. Enhanced subnational engagement and empowerment can build ownership, improve programme alignment with local needs and foster sustainable, community-driven implementation.

**HOW THIS STUDY MIGHT AFFECT RESEARCH, PRACTICE OR POLICY**

- Governments and policy-makers need to prioritise domestic resource mobilisation and increase allocations for HIV prevention funding into national health budgets to reduce reliance on donor funding and ensure sustainability.
- Research on the effects of staff motivation and retention initiatives, including both financial and non-financial incentives, would provide valuable insights for workforce management in health systems.
- Systematic involvement of subnational stakeholders in programme planning and governance/decision-making is essential for fostering ownership, aligning programmes with local needs and achieving programme integration and sustainability.

## Background

 HIV is the leading cause of death and disability-adjusted life-years in Zimbabwe.^1^ Zimbabwe has 1.3 million adults and children living with HIV as of 2024.^2^ WHO and UNAIDS designated Zimbabwe as a priority country for voluntary medical male circumcision (VMMC), due to unprotected heterosexual sex as a driver of HIV transmission.^2 3^ The sustainability of the HIV prevention programme, of which VMMC is a part, is critical for ending the HIV epidemic in Zimbabwe. VMMC can reduce a male’s heterosexual acquisition of HIV by 60% through a one-time procedure that provides lifelong prevention benefits.^4^ Additionally, VMMC can reduce the risk of STIs and penile and prostate cancer in males and the risk of sexually transmitted infections (STIs) and cervical cancer in females.^5^,^8^

**Figure 1:**
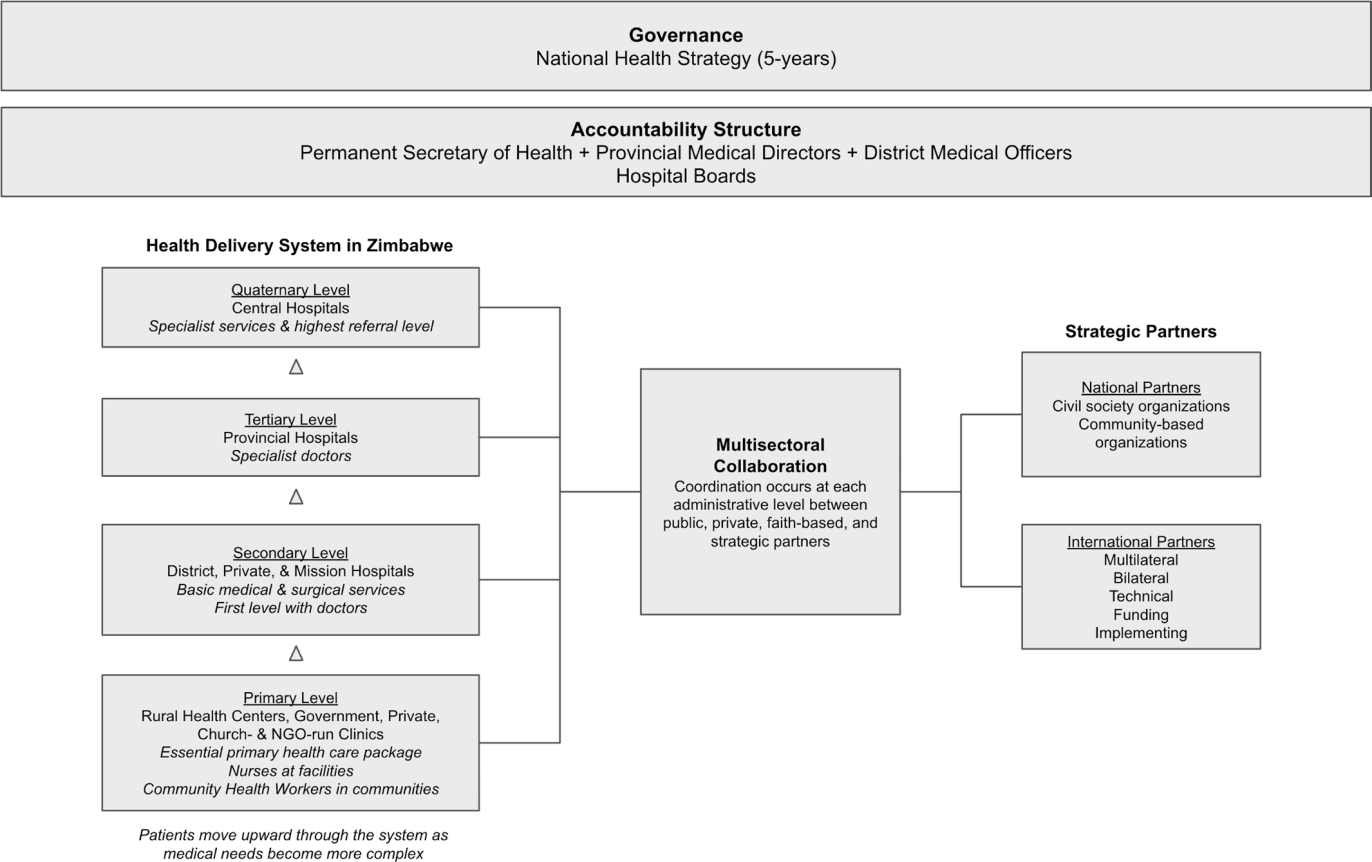
Zimbabwe healthcare system. NGO, non-governmental organization.

As part of the national HIV strategy, VMMC was introduced in 2009 as a vertical programme, with a focus on a single disease, driven by external donors and implementing partners.^9 10^ It is available at primary and secondary level static sites, where trained providers are stationed to do circumcisions independently or with support from a secondary level team, or performed by a roving team, at a caravan or tent set up as a temporary operating theatre. The tertiary level handles severe adverse events and provides surgical mentorship support to provinces. A total of 1127 facilities in all 64 districts in Zimbabwe provide VMMC. The Ministry of Health and Child Care (MoHCC) oversees provision of VMMC services through a devolved structure with technical and logistical support from a national level steering committee. While most circumcisions are done by nurses, some doctors perform this service at district level, in addition to managing adverse events and serving as backup to nurses. The entire programme has not yet switched to results-based financing from a fee-for-service model. See figure 1 for more details on the structure and governance of the healthcare system in Zimbabwe.

The vertical approach worked for launching and scaling the programme. However, vertical, donor-dependent health programmes in low-income and middle-income countries face the risk of discontinuation when external funding is withdrawn, if they are not integrated into the broader health system. Of the total health budget for Zimbabwe, 35% came from foreign assistance in 2023, and the US government funded 80% of the US$250 million budget for HIV and STIs (in an email from Sinokuthemba Xaba (xabasino@gmail.com) in April 2025.)^11^ Although VMMC had secure donor funding that decreased over time and was projected until at least 2027, this funding was abruptly withdrawn with the end to US foreign aid.^12 13^ According to the WHO, ‘in the integrated model (a.k.a horizontal), services do not have separate administration or budgets and are typically delivered through health facilities that provide routine or general health services.’^14^ The MoHCC committed to transitioning the VMMC programme to an integrated programme, guided by the Sustainability Transition Implementation Plan, 2019–2021.^9^ Ideally, this plan would have been created when the programme was initially introduced.

The transition of VMMC services, or any vertically funded programme, in Zimbabwe from a donor-funded to government-owned and operated programme requires the collective efforts and alignment of various stakeholders: national and provincial government leaders, district managers, healthcare providers, village health workers, community members, donors and non-governmental organisations. This transition is particularly difficult due to the unstable political and economic situation in the country and is still ongoing. Staff retention is a major issue as trained staff leave for jobs outside of the government or emigrate.^15 16^ Additionally, the prevailing macroeconomic challenges, hyperinflation and currency changes, affect health workers’ earnings and working environments.^17 18^

The description of India’s HIV programme transition to government ownership is one of the few studies detailing the specific processes that contribute to the successful transfer of ownership and integration of a large-scale, donor-funded vertical health programme.^19^ In this case, the government of India was poised to take full financial responsibility for the HIV programme. Vu *et al* also described the transition of the VMMC programme in PEPFAR-funded districts in Zimbabwe from management by a USA-based organisation to a private local organisation.^20^

The aim of the study in this paper was to understand and examine the perspectives of individuals in various roles at multiple levels regarding the barriers, facilitators and recommendations for transitioning an HIV prevention programme, and the VMMC programme specifically, from donor-led to government-owned and operated. This study is not an evaluation of the transition process. The research was situated within a larger intervention called ‘OPTIMISE’ using the Leadership and Engagement for Improved Accountability and Delivery of Services (LEAD) Framework (described elsewhere) to further inform the sustainable integration of the VMMC programme into routine health services in Zimbabwe.^21^ The overall goals of the OPTIMISE project were to: (1) inform the transformation of HIV prevention into a sustainable programme, with a focus on integrating the VMMC programme into mainstream health services and (2) support and capacitate the MoHCC in working with stakeholders to develop and implement sustainability plans.

## Methods

### Study design

Due to the time-limited nature of the project and research question, the team conducted a cross-sectional qualitative study to gather stakeholder perspectives from multiple levels of the health system to feed into and inform implementation of the LEAD Framework within the OPTIMISE project. The LEAD Framework is a bottom-up systems change approach that empowers teams to identify, prioritise and resolve operational challenges. The research team had demonstrated its effectiveness in strengthening leadership and management capacity in malaria programmes in Eswatini, Namibia and Zimbabwe.^22^,^24^

The research team was led by the principal investigator, who collaborated with Zimbabwean independent consultants and MoHCC representatives, two of whom were co-PIs of the study. Their contributions included the formulation of research questions, selection of methods and data collection. Additionally, implementing partners in Zimbabwe provided valuable input to the analysis and interpretation of results (online supplemental file 1). After pretesting the interview guide, a local market research firm conducted 51 of the interviews while AMC and NB conducted the remainder.

### Recruitment

We used purposeful sampling, interviewing key informants about the subject until theoretical saturation was reached.^25 26^ Individuals were identified in close consultation with Provincial Medical Directors and then formally invited to take part in the study. All participants were selected for their first-hand knowledge of the topics covered during the interviews and had roles that were directly involved in governing the VMMC programme, delivering services or promoting the programme through community outreach. No one declined to participate. ‘Saturation’ occurred when the team had completed several iterations of analysis and found that the data no longer yielded additional new themes or insights. Interviews at the subnational level included participants in five districts, who received invitation letters from the MoHCC. Most national level interviews were conducted in Harare.

### Data collection procedures

We used separate interview guides for different stakeholders, which were piloted before use with two individuals occupying different roles within the programme. The team adjusted the guides to avoid unclear wording and biases and to ensure questions accurately reflected the intended topics. All interviews took place between April 2021 and February 2022 and were done in English, which was spoken and understood fluently by the sampled respondents. While the local research team did most interviews face to face, seven were conducted by phone or videoconference by AMC and NB. Interviews ranged from 20 min to 90 min. Audio recordings of all interviews were transcribed for analysis. All data were saved on a secure, password-protected cloud-based platform to which only research team members had access.

### Data collection domains

We used eight domains in the interview guides, which were adapted from the WHO health system building blocks. The domains are listed and defined in table 1.

**Table 1:** Interview guide domains and topics

Domain	Topics covered by domain
Transition of programme	How VMMC programme components financed, managed and operated by external partners will be transferred to government
Integration	Current state of integrating VMMC into routine health services, lessons learnt from other efforts, current/potential challenges and mitigation strategies
Decentralisation	Challenges and mitigation strategies for improving the success of the VMMC programme in the context of decentralisation
Financing	Challenges from declining donor support for the programme, mitigation strategies for ensuring the financial sustainability of integrated VMMC services
Human resources	Challenges, motivation, incentives and recommendations to address attrition, capacity gaps
Community engagement	How the programme works with the community to ensure uptake and acceptability of VMMC services, perceptions, experience, barriers, challenges, recommendations
Quality assurance	Concerns about the impact of transition process on quality and accessibility of VMMC services, how to address concerns
Health information	Current gaps in VMMC data systems, changes to ensure the effectiveness and sustainability of information systems

VMMC, voluntary medical male circumcision.

### Analysis approach

We used the framework method to analyse the interview data.^27^ The data analysis team of three individuals used the audio recordings and the transcripts to familiarise themselves with the data. The team worked independently to manually code the transcripts, employing a hybrid approach that incorporated deductive and inductive codes.^28^ No software was used to assist with coding. Subsequently, the team met to compare the codes they had applied, ensure coding consistency and develop an evolving codebook with codes that emerged from the data. At this stage, coders interpreted the data by analysing derived themes and, where appropriate, making connections with categories already established in secondary literature. Finally, the team compiled findings in a summary document organised by themes with illustrative quotes.

### Validation approaches

To enhance the validity and credibility of the data analysis, we presented preliminary findings by theme with illustrative quotes through member checks with interview participants and other stakeholders.^29 30^ These in-person workshops, held in April 2022, included stakeholders at the community, facility, district, provincial and national levels. We made minor adjustments to the representation of some concepts at that time. We conducted additional member checking with the MoHCC and implementing partners in June–July 2022. We also validated findings and incorporated multilevel stakeholder feedback during final study workshops in September 2022.

## Results

### Study participants

54 individuals participated in the semistructured in-depth interviews: 43 stakeholders from various levels within the health system and 11 interviews with national-level stakeholders (table 2). Most of our interviewees were male (70%). Participants were equally distributed across the selected provinces and represented perspectives across varying levels and roles within the health system. These roles included funding and managing the VMMC programme, performing circumcisions, training circumcisers, educating and generating demand among communities, collecting and analysing data and ensuring adequate supplies and equipment. Many of these responsibilities were not exclusive to the HIV programme but extended to the entire health system.

**Table 2:** Role designations and number of interview respondents per role

National roles	Provincial roles	District roles	Facility/community roles
Ministry of Health (4)	Provincial medical director (2)	District medical officer (4)	Nurse circumciser/nurse in charge (4)
Technical/implementing partners/donors (7)	Provincial VMMC officer (1)	District nursing officer (4)	Pastor (1)
	Provincial maternal and child health officer (2)	District health information officer/assistant (4)	Chief (1)
	Provincial education officer (1)	District schools inspector (4)	
	Provincial HIV focal person (1)	District team leader/officer (2)	
		District health services administrator (1)	
		District VMMC focal person (3)	
		District health promotion officer (2)	
		District pharmacy manager/technician (3)	
		District AIDS coordinator (3)	

VMMC, voluntary medical male circumcision.

### Barriers, facilitators and recommendations for integrating and sustaining VMMC

We structured our analysis in line with the WHO health system building blocks framework, which remains valuable when used as a descriptive scaffold that acknowledges the dynamic interactions between system components while also considering communication, social determinants of health, equity and health system resilience. All these factors arose during focus group discussions and our intervention to integrate VMMC into routine health services. The findings on health system resilience and communication are covered in another article,^21^ and results related to equity and social determinants of health from focus groups were shared with the programme and community through other channels best suited to reaching those audiences.

**Figure 2:**
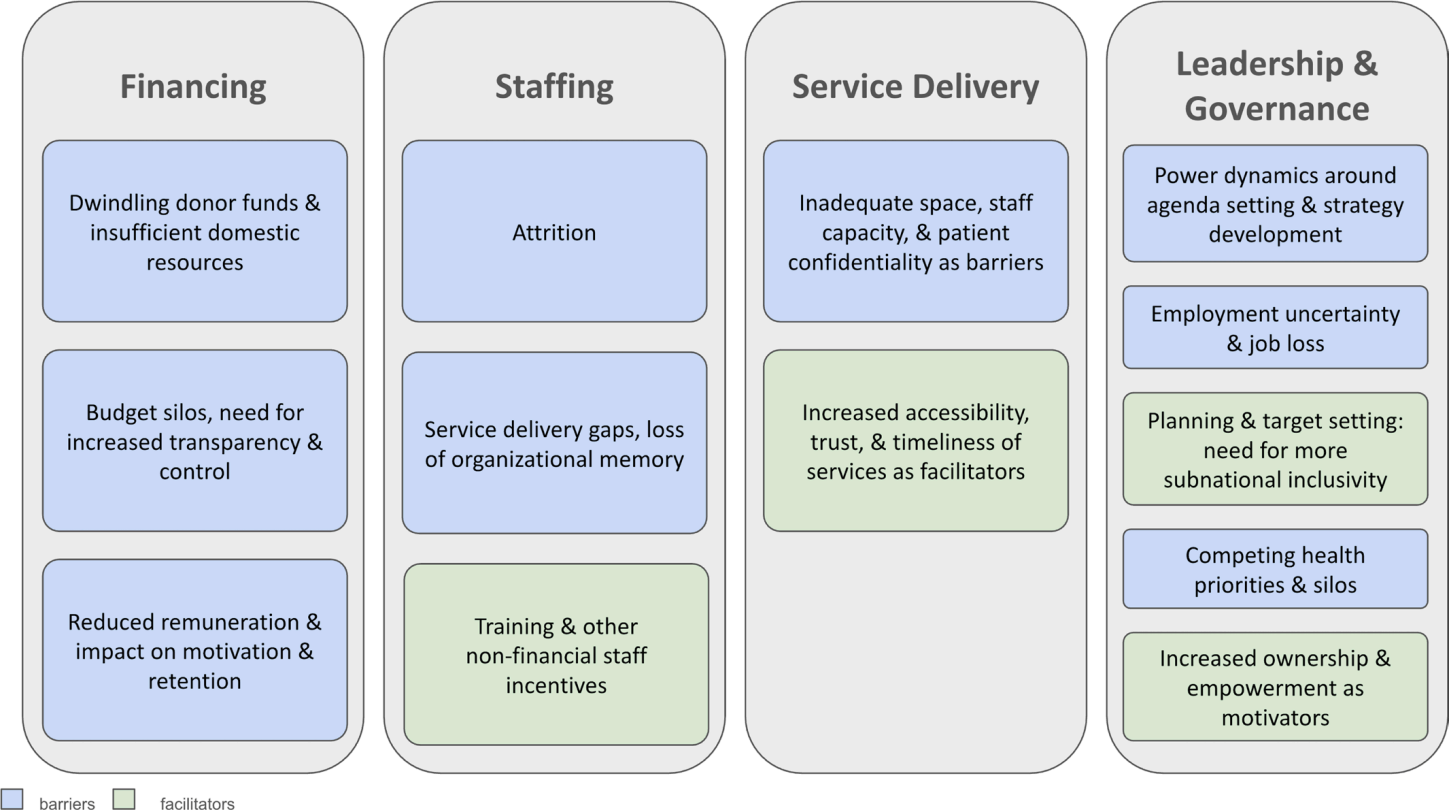
Themes and categories.

We identified four major themes affecting the sustainability and integration of VMMC services within routine clinical care: (1) financing, (2) staffing, (3) service delivery and (4) leadership and governance (figure 2). These domains include psychological and structural barriers and facilitators. While the WHO building blocks were used as organising categories, additional themes arose that cut across building blocks. These included decentralisation, motivations and incentives, collaboration and coordination, and equity (in pay and service availability), which we describe in further detail below. Additionally, challenges arose related to medicines and information systems that were not unique to the VMMC programme. We present findings in order of their relevance to integrating and sustaining VMMC. All illustrative quotes contain an anonymised respondent identifier and indication of gender (M/F). We found substantial alignment between the respondents and the strategies devised by district health teams during project workshops conducted in April 2022. However, the concerns around job security for implementing partners and the influence that donors and partners have on the programme due to their control of the financing were sensitive topics that individuals only shared in confidence during interviews.

To further triangulate the data, we conducted a structured document review, analysing the challenges and solutions within the five study intervention district work plans generated through the LEAD Framework.^29 30^ The work plans were developed by district ‘task teams’—groups established to address and resolve challenges to integration and sustainability of the VMMC programme. We compared the data in the work plans to themes arising from the interviews. The significant overlap between the work plan inputs and the interviews helped to corroborate our findings. More details on this review can be found in another article.^21^

### Financing

Financing challenges, particularly concerning domestic resource mobilisation, budget silos and transparency, and remuneration, impact the sustainability of the VMMC programme at the programme and provider levels.

#### Dwindling donor funds and insufficient domestic funds

Most participants understood that a critical focus for any sustainable programme is the replacement of donor funds with domestic resources. Shifting the programme from its current reliance on external donors is a long-term goal that will move the programme towards sustainability. As one district officer observed:

As for now it is mainly partner supported. So in the absence of the partner, it can be tricky to sustain the gains we have realized so far. The ultimate call for the MOH is to have complete ownership and prioritizing funding for VMMC. (P20, M)

Respondents expressed concerns about insufficient local funding for VMMC, with the underfunding of the national health budget, and most of the HIV budget going towards treatment rather than prevention.^31^ Some participants worried about the need for trade-offs with other health priorities, given the finite amount of health financing. To address these challenges, recommendations included incorporating VMMC into results-based financing and mobilising domestic resources. A provincial officer’s remarks typify how respondents view the issue:

We need a sustainable way to actually fund our VMMC activities. This may probably mean that the source of funding should be from government as opposed to donor funds. (P24,M)

#### Budget silos and the need for increased transparency and control

Although not as frequently raised as concerns around the withdrawal of donor funds, disease-specific funding tended to create silos rather than encourage integrated planning. Participants wanted to see greater alignment across national-level strategic plans and donor investments. They also suggested that implementing partners may not be motivated to integrate, as it could result in a loss of control over financing, a point captured well by one of the implementing partner staff members we interviewed:

It’s frustrating when you're asked to do things, and you don't see the purse [money]. You don't have control of the purse. It’s hard to implement. But when you are holding the purse, you are able to drive your team. So the money will be the big game changer; how it moves. (P46, M)

#### Reduced remuneration and the impact on motivation and retention

In addition to the programme financing challenges, many respondents highlighted the evolving nature of remuneration and its impact on individual providers. Initially, providers were compensated on a per-procedure basis, which allowed them to supplement their regular incomes. Several respondents expressed concerns that the reduction in financial incentives for VMMC services could impact staff motivation and retention, as noted by a district officer:

When the program started, the cost-benefit reimbursement was quite good. A lot of people benefitted from this program. So as we move towards sustainability, that means the incentive may not be there … It requires higher authorities to ensure that they appreciate the work that’s being done by health workers, and their remuneration is one of the most serious things, because if people have got good remuneration, they rarely move from one place to another*.* (P13, M)

Although most respondents acknowledged providers should not continue to be financially incentivised for performing VMMC, at least one district officer commented:

I think the financial aspect is the key, looking at the economic environment that we are in. Everyone is looking for greener pastures. The financial aspect is key to motivate staff to take on new challenges*.* (P18, F)

### Staffing

A second major domain of structural challenges to integration reported by most participants pertained to staffing shortages due to attrition, which create service delivery gaps and loss of organisational memory. To motivate and retain staff, respondents had recommendations related to training and other non-financial incentives.

#### Staffing shortages from attrition and subsequent consequences

Health workers leave government jobs for various reasons, including the poor economic situation, low wages, delays in receiving payment and under-resourced working conditions. These factors were widespread issues throughout the health system and not unique to Zimbabwe. Ensuring the availability of VMMC services at lower-level facilities entails retaining an adequate pool of circumcisers and trainers. Training circumcisers requires a substantial investment in funding and time. Additionally, there is the opportunity cost of having fewer providers to serve clients while they undergo training. One district officer summarised staff attrition challenges succinctly as follows:

The Ministry and the partners have invested a lot of money in training those people. The reasons given [during] the exit interview should be used as a baseline for coming up with ways of dealing with staff attrition. Mainly it’s on the financial aspects, people are looking for places where they can actually survive and keep their families. (P18, F)

#### Service delivery gaps and loss of organisational memory

Participants pointed out various negative impacts of staff attrition, such as the inability to accommodate walk-in VMMC clients. Additionally, the health system suffers from the loss of organisational memory when experienced staff members depart. Consider, for example, this observation offered by a district officer:

I will still go back to the fact that the facilities do not have trained cadres, which means clients are going, but they are not being circumcised. (P23, M)

#### Training and other non-financial incentives

To help facilitate programme integration among healthcare providers, some respondents mentioned non-financial incentives, such as training, a sense of purpose and contributing to reduced disease burden, as key motivators for staff that could also promote retention. For example, a provincial officer made the following points:

What also incentivizes health care workers is a well-run program … People get discouraged once consumables are not readily available or once results are not being met. But if the program runs well with targets being achieved, and being celebrated, the health care workers appreciate it. (P24,M)

### Service delivery

A transition to sustainability will also involve decentralisation of service delivery to primary healthcare facilities, which will be challenging due to limited space and staff capacity. If the programme can address these challenges, increased accessibility, trust and timeliness of services are facilitators for programme integration that could make it more patient-centred.

#### Inadequate space, staff capacity and patient confidentiality as barriers

Subnational leaders consider catchment areas and coverage rates in deciding which primary health facilities should offer VMMC. Respondents raised insufficient space and the challenge of maintaining sterility as obstacles to service delivery at lower-level facilities. Given that the procedure involves the removal of the penile foreskin, the provider needs ample space to ensure comfort and privacy. One implementing partner, for instance, identified the dilemmas faced in practice:

So apart from staff, the other shortage is space, especially at our local clinics … Now you are adding a VMMC service to a 2-room clinic. Our infrastructures were built for a few health programs, but over the years … other health programs … have come and recently there’s COVID, where you need a dedicated room. So imagine you are just adding more and more programs to a fixed site. Where would these programs be taking place? (P12, M)

Participants also mentioned limited staff capacity as a barrier to decentralisation of services. The issue is summarised well by a district officer we interviewed:

In May we were doing national immunization, HPV, PCV and so forth. You find most of our health workers were taken to do that but at the same time we need those health workers to do VMMC, so that could be a challenge. (P14, M)

Furthermore, service providers will need to take on these additional responsibilities without increased compensation, as the programme cannot continue to pay per procedure. As one implementing partner stated*:*

…you are also adding responsibility to a worker who is already overburdened and also does not have adequate remuneration. Unfortunately, the economic environment has not been conducive because of the poor remuneration, so it’s like adding burden to someone already earning less. (P12, M)

Some respondents expressed concerns about provider-patient confidentiality in the context of decentralisation. They raised the possibility of reduced privacy when clients and providers live in close-knit settings. This privacy and confidentiality challenge is well exemplified by an implementing partner:

So the moment we decentralize, or we say we are doing it at clinic level, if I am a nurse at the clinic, I know John, Peter, and Stephen and know where they stay. The moment I circumcise them, they will think that I will tell everyone that I have circumcised them. (P36, M)

#### Increased accessibility, trust and timeliness of services as facilitators

While decentralisation presents staff at facilities with concerns around confidentiality and privacy, other respondents suggested that implementing these changes could yield several advantages for patients: enhanced accessibility, increased trust and timeliness of services, and a reduction in waiting times and referrals. Consider, for instance, these remarks by a district nurse regarding issues of trust:

This may make the numbers increase…people at a certain area… feel secure, if they are dealing with the nurses they know … They don't normally trust people, whom they think are strangers. They want their local people. They [are] listening better to their local people than to see a new face coming to start talking about VMMC. Decentralization … will help. (P4, F)

### Leadership and governance

A lack of alignment around ownership of the programme, with disagreement around whether the government or funders and implementing partners held the power, was widely perceived as a significant barrier to programme transition. Other barriers included employment uncertainty, top-down planning and competing health programmes. Increasing ownership by subnational stakeholders was a facilitator that should be leveraged.

#### Power dynamics around agenda setting and strategy development

The main goal of the transition to sustainability is government ownership and operation of the programme at national and subnational levels. A major obstacle influencing organisational readiness for change is that participants disagreed about who currently holds the power to lead the programme, such as setting the agenda and developing strategies. A ministry-level respondent asserted the government’s perspective:

The Ministry [sets the agenda by] doing the HIV estimates. It also does the Demographic Health Survey, which is done every four years. So on an annual basis, the government sets what the needs are and then presents this to partners. (P48, M)

For some, the MoHCC has the power to set the direction of the programme. Others perceived a power asymmetry between the MoHCC, donors and partners. These individuals equated the control of money that partners and donors have with the ability to influence programme strategies. Foreign aid is funnelled to implementing partners, rather than given directly to the government.^32^ Given this arrangement, external donors have an interest in seeing how their funds are used so they are not diverted to other areas. The following quote from an implementing partner illustrates this differing perception:

[Donors] have a hand in how the country strategies are shaped because after funding, they are actively involved in the implementation of the grant, so development of strategies. (P47, M)

According to respondents, the availability of funds for regular convening to discuss programme-related matters plays a pivotal role in subnational ownership by management structures, such as Provincial or District Health Executives (PHE/DHEs). Influence over setting the agenda and driving the programme also plays out at the subnational level. The MoHCC sets the agenda for part of the meeting, and partners control the rest of the agenda. The same implementing partner captured current power asymmetries regarding agenda setting:

There is supposed to be a subnational level provincial health team meeting four times a year. It rarely ever happens unless it’s funded by someone who determined the agenda of that meeting. At district level, they're supposed to have bi-annual district health team meetings. They rarely ever happen, and when they happen, they are funded by someone who determines the agenda. (P47, M)

#### Employment uncertainty and job loss

In addition to the desire of donors to oversee how their funds are used, employment uncertainty may influence the decision-making and actions of implementing partners. Resistance to the transfer of programme control from partners to the government may be connected to the influence of change valence, the degree to which organisational members deem the change to be beneficial, worthwhile, important or necessary, especially when a programme transition would result in the loss of their jobs.^33^ Consider, for example, this observation on the part of a donor we interviewed:

I think that what ends up being a challenge a lot of times is that, when you say working yourself out of a job, it actually means some individuals might actually not have a job anymore because [the partner is] no longer funded to be doing the thing that they were doing before. And I think that doesn't actually sit well. (P45, F)

#### Planning and target setting: need for more subnational inclusivity

With the shift towards government ownership, subnational stakeholders will take on increased leadership and management responsibilities. Participants highlighted limited local engagement by donors and partners in planning and policy development as a major obstacle. These processes are currently conducted in a top-down manner that could be more inclusive, incorporating the needs and priorities of local communities. This normative point was made strongly by a provincial officer who asserted:

Sometimes [implementing partners] do not plan with the Provincial Health Executive (PHE). They plan with their [satellite] office without planning with the PHE. So they have to plan together and… come on board to solve our problems because we have to identify the gaps and fix and cover the gaps together. (P30, M)

Agenda and target setting should also aim to be more inclusive of subnational stakeholders, such as the PHEs, DHEs, health facilities, community-based organisations and local leadership. Some participants emphasised the need for district level targets, in addition to provincial level targets set at national level. A normative recommendation in this regard was made by a district officer we interviewed:

The partners and the Ministry … try to engage [stakeholders], but …they need to engage them on a quarterly basis or … monthly basis to come … to the communities that we serve. They [could] …interview local leadership to find out how best it can be implemented and … the service provider for service delivery and find out how best the program can be managed. (P18, F)

#### Competing health priorities and silos

Vertical funding and organisational structures have contributed to a mindset that integrating and sustaining the VMMC programme may negatively impact other health programmes. Respondents noted that VMMC is not always prioritised by frontline workers or seen as a key component of HIV prevention. For instance, this district nurse observed:

People may not fully appreciate the role of VMMC in HIV prevention. They might want to sideline it and give much more time in the focus on other departments and programs. (P2, M)

#### Increased programme ownership and empowerment as motivators

By engaging local stakeholders in the programme’s governance, the Ministry has a valuable opportunity to enhance ownership of the VMMC programme and empower subnational stakeholders. This empowerment can be highly motivating, as it instils a sense of responsibility for the positive changes they enact and the tangible improvements resulting from their decisions and actions. The value of empowerment was expressed clearly by this implementing partner we interviewed:

I've seen people are supported … where districts and facilities own or identify the challenges that they're facing, and prepare plans to turn around those challenges, and track certain indicators on a regular basis … And they're actually owning those changes, in terms of the numbers changing in the direction that they were looking for. And the more the staff see improvements, they feel more motivated actually to do better, using their data for decision making. (P46, M) 

Despite the additional burden of taking on more responsibilities, subnational interviewees would be receptive to being more accountable for the programme. In fact, perceptions about the programme might change from being perceived as partner-driven to community-owned, as exemplified by a provincial officer and provincial-level trainer we interviewed:

We are ready. It’s not like right now we're not doing anything … [Decentralisation] will actually empower us more and allow us to apply things that are applicable and relevant to our provinces. (P10, M)

We want the community to own [the program] … We want them to feel that they are part and parcel of this program and move away from thinking it’s partner funded and everything, but we want them to actually own it and support it. (P31, F)

## Discussion

This study investigated stakeholder perspectives on transitioning the VMMC programme from donor-led to government ownership. Respondents identified key structural barriers, including financing and staffing issues, and expressed concerns about the implications of donor funding withdrawal without adequate domestic resources. The shift from fee-for-service to results-based financing that rewards facilities adversely impacted individual providers reliant on this income. Additional psychological barriers included job loss for some, increased responsibilities for others and disagreement over decision-making authority between donors, partners and the government. Although attrition hindered service decentralisation, the transition offered opportunities for greater ownership among subnational stakeholders and improved client access, trust and timeliness. Addressing these barriers and leveraging facilitators will be essential for ensuring a smooth transition of this vertical HIV prevention programme.

Our findings relating to structural and psychological barriers and facilitators align with experiences in other regions. A study in Cambodia and Pakistan found that donors influence priority setting and the entire policy process through their control of financial resources.^34^ The VMMC programme has not reached a comparable stage as the India HIV programme, due to the challenges of channelling donor funds to the government and generating sufficient domestic resources.^19^ Our results align with Weiner’s theory of organisational readiness for change, which emphasises the importance of psychological factors in influencing organisational members’ shared change commitment and collective judgement about their capacity to enact change.^33^ Additionally, our findings with respect to staffing echo the global phenomenon of brain drain, where additional training facilitates health workers’ migration to high-income nations.^35^ Factors contributing to the persistence of vertical HIV clinics in Uganda include a shortage of trained staff, insufficient space and an increased workload on health workers.^36^ Moreover, a study in Zimbabwe showed that individual providers were more motivated when paid per circumcision, leading to rapid achievement of performance targets.^37^ Finally, a study of the sustainability of US government-funded health projects in five countries found that unstable political and economic conditions in Africa impeded sustainability.^38^

Our study differed from others by incorporating perspectives from a diverse set of stakeholders across multiple levels of the health system. A key finding of our study was a lack of consensus among these stakeholders regarding the leadership and oversight of the programme. Opinions diverged significantly, with some stakeholders attributing governance to the government, while others placed it with donors or implementing partners. Notably, these differences were evident even within the same stakeholder group (eg, implementing partners). In contrast to much of the existing research on programme integration and sustainability, which focuses on service delivery, our study underscores the significance of contextual factors, such as financial organisation and accountability, staff motivation and retention, and leadership and governance.^39^,^41^

Implications of our work include the need for preservice and in-service training to ensure a sufficient pool of trained staff. Decentralisation of governance represents an opportunity to foster a deeper sense of ownership among subnational stakeholders for managing health services while also allowing for tailoring of the programme to local conditions. Involving subnational entities in the governance of the programme can motivate them and cultivate accountability for its success. With limited health budgets, governments should evaluate the cost-effectiveness of different interventions against HIV. They need to also determine the optimal combination of interventions for specific populations and assess how these interventions impact existing health systems by supporting or weakening them, and explore ways in which new interventions can better benefit health systems overall. Engaging in supra-national collaboration with donors to reconfigure the operating model holds the potential to mitigate the issue of fragmented, disease-specific donor financing, which often neglects health system strengthening. A shift of focus towards incorporating local needs into programme planning is also imperative, ensuring that initiatives are driven by district and community priorities. The current state of the HIV prevention programme in Zimbabwe leaves it vulnerable. Without adequate domestic resources and political commitment in place to bridge the gap, there is a significant risk of programme discontinuation now that donor funding has been severely compromised.

Further research is needed to investigate the feasibility of mobilising domestic resources, particularly within the challenging context of an unstable political and economic climate. In Zimbabwe, it is important to assess and monitor the impact of withdrawing donor support, especially whether districts can maintain their performance targets in the absence of donor funding, as this will provide valuable insights into programme sustainability and health system resilience. To tackle attrition and retention, research should identify strategies that can effectively mitigate the brain drain and incentivise healthcare personnel to accept and remain in government positions. Such research is crucial for addressing the persistent shortage of healthcare workers in Zimbabwe and other low-income and middle-income countries.

We acknowledge several limitations in our study design. First, our interviews were confined to five districts where we implemented our project and may not be generalisable to the entire country. Second, most respondents were actively engaged in our intervention, introducing uncertainty regarding how their participation may have influenced their responses. Third, the research team did not consistently probe during the interviews. Finally, qualitative data analysis inherently relies on interpretation by individuals closely engaged with the data. We mitigated this subjectivity by having three individuals code and analyse the data and validated results with stakeholders at various stages to improve representativeness of the findings. Despite these limitations, results provide critical insights into integration and sustainability of HIV prevention in Zimbabwe.

## Conclusions

Integration is a way to enhance effectiveness, sustainability and national autonomy, but it challenges the status quo, including the role and power of aid donors, control and accountability of donated funds, and subnational policy and governance. For the HIV prevention programme to achieve integration and sustainability, it must address both psychological and structural barriers associated with these challenges. Donors need assurance that the government will institutionalise VMMC into routine health services and that it will be fully accountable for funding these services. Decentralisation of VMMC to lower-level facilities will be challenging, particularly if staff attrition remains a major obstacle. The programme should leverage opportunities that will facilitate this transition, making it more patient-centred and connected to the community. Zimbabwe’s experience in navigating the transition of its vertical health programme can serve as a valuable case study for other low-income and middle-income countries undergoing similar shifts. Indeed, the salience of this study is all the greater considering the recent withdrawal of development assistance for health globally. By learning from Zimbabwe’s experiences and challenges, other countries may glean insights into how to effectively manage the transition toward integrated and sustainable healthcare programmes.

## Supplementary material

10.1136/bmjgh-2024-018732 online supplemental file 1

## Data Availability

Data are available on reasonable request.
