# Supplementary material for: Understanding stakeholder perspectives on integrating and sustaining a vertical HIV prevention programme into routine health services in Zimbabwe: a qualitative study
Source: BMJ Glob Health. 2025 Aug 11;10(8):e018732. doi: 10.1136/bmjgh-2024-018732 (PMC12352186; doi:10.1136/bmjgh-2024-018732)
Supplement: online supplemental file 1 [file bmjgh-10-8-s001.pdf]

Supplementary File 1: Reflexivity Statement

1. How does this study address local research and policy priorities?

This study was conceptualized to address local research and policy priorities in partnership with the Ministry of Health and Child Care (MoHCC) of Zimbabwe.

2. How were local researchers involved in study design?

Co-Principal Investigators, including representatives from the MoHCC (OM) and the LEAD Framework team (JM), designed the study, providing input into the interview guides and participant list.

3. How has funding been used to support the local research team(s)?

Funding was used to support the salaries of the local research team and a contract with a local market research company.

4. How are research staff who conducted data collection acknowledged?

Research staff who conducted data collection are acknowledged by name in the acknowledgment section.

5. How have members of the research partnership been provided with access to study data?

All transcripts have been saved in an open access repository.

6. How were data used to develop analytical skills within the partnership?

All participants were involved in the data analysis at in-person workshops.

7. How have research partners collaborated in interpreting study data?

Data were shared with respondents for validation, and research partners were consulted regularly to ensure accuracy of data interpretation.

8. How were research partners supported to develop writing skills?

Research partners were involved in drafting and revising the manuscript.

9. How will research products be shared to address local needs?

Focus group findings were shared as personas to inform the work of MoHCC VMMC Advocacy and Communications Technical Working Group and key informant interview findings were shared as presentations during midline and transition workshops.

10. How is the leadership, contribution and ownership of this work by LMIC researchers recognised within the authorship?

LMIC researchers (JM, PCh, RC, SX, GN, OM, PK) have been recognized as co-authors for their leadership, contributions, and ownership of this work.

11. How have early career researchers across the partnership been included within the authorship team?

Early career researchers (AMC, JM) were included within the authorship team. They were involved throughout the research and writing process. We acknowledge that AMC is based in a high-income country.

12. How has gender balance been addressed within the authorship?

Six authors are female (AMC, PCh, RC, GN, PK, NP) and seven authors are male (JM, PCa, JG, SX, OM, RG and SMB). We acknowledge that we could have a better gender balance on the authorship team.

13. How has the project contributed to training of LMIC researchers?

The larger project includes research led by JM, who will also be first author on writing a manuscript to summarize the results. Follow-on funding of the larger project was awarded to a local Zimbabwean organization run by PCh. These are important steps in ensuring more equitable research partnerships.

14. How has the project contributed to improvements in local infrastructure?

The larger project has not directly contributed to improvements in local infrastructure but it did strengthen leadership and management capacity among local stakeholders and created a cadre of trained facilitators who received a Professional Practice in Change Leadership certification from the University of West of England (UWE) and a new partnership between UWE and Women's University in Africa to deliver this training.

15. What safeguarding procedures were used to protect local study participants and researchers?

There were multiple safeguards and ethical approvals obtained and training conducted with researchers to ensure confidentiality and privacy in regards to participant participation and data.
